# Sharp maximal and weighted estimates for multilinear iterated commutators of multilinear integrals with generalized kernels

**DOI:** 10.1186/s13660-017-1553-2

**Published:** 2017-11-07

**Authors:** Yan Lin, Nan Zhang

**Affiliations:** School of Sciences, China University of Mining and Technology, Beijing, 100083 P.R. China

**Keywords:** 42B20, 42B25, 42B35, multilinear singular integral operator, multilinear iterated commutator, $BMO$ function, sharp maximal function

## Abstract

In this paper, the authors establish the sharp maximal estimates for the multilinear iterated commutators generated by $BMO$ functions and multilinear singular integral operators with generalized kernels. As applications, the boundedness of this kind of multilinear iterated commutators on the product of weighted Lebesgue spaces and the product of variable exponent Lebesgue spaces can be obtained, respectively.

## Introduction

The multilinear singular integral operator theory plays an important role in the singular integral operator theory of harmonic analysis. On the one hand, many researchers have put plenty time and energy into this topic. Kenig and Stein did many works on the multilinear fractional integral operator in [1]. Grafakos and Torres established the multilinear Calderón-Zygmund theory in [2]. The boundedness of multilinear Calderón-Zygmund operators with kernel of Dini’s type was studied by Lu and Zhang in [3].

On the other hand, more and more researchers have been interested in multilinear commutators. The multilinear commutator was given by Pérez and Trujillo-González in [4]. The weighted estimate for multilinear iterated commutators of multilinear fractional integrals was studied by Si and Lu in [5].

Some useful conclusions of multilinear singular integral operators with generalized kernels were given by Lin and Xiao in [6]. With more weaker conditions for the kernel, they got the conclusion on sharp maximal estimates of the multilinear singular integral operators and their multilinear commutators with $BMO$ functions. Moreover, the boundedness of the multilinear commutators with $BMO$ functions on the product of weighted Lebesgue spaces and the product of variable exponent Lebesgue spaces was acquired in [6] as well.

Pérez, Pradolini, Torres and Trujillo-González studied the multilinear iterated commutators of multilinear singular integrals with Calderón-Zygmund kernels in [7]. They first established the sharp maximal estimates, then the end-point estimates were acquired.

Based on these studies above, we will focus on the multilinear iterated commutators of multilinear singular integrals with generalized kernels in this paper. And we will consequently establish three theorems as conclusions in Section 2. First, the sharp maximal estimates of multilinear iterated commutators generated by $BMO$ functions and multilinear singular integral operators with generalized kernels will be established in Theorem 2.1. Then, the boundedness of this kind of multilinear iterated commutators on the product of weighted Lebesgue spaces and the product of variable exponent Lebesgue spaces will be put forward by Theorems 2.2 and 2.3, respectively. In addition there are some necessary lemmas in Section 3. The proof of the main results will be given in Section 4.

Let us recall some necessary definitions and notations firstly before starting our main results.

### Definition 1.1

([6])

Let $m\in \mathcal{N}_{+}$ and $K(y_{0},y_{1},y_{2},\ldots,y_{m})$ be a function away from the diagonal $y_{0}=y_{1}=\cdots =y_{m}$ in $(\mathbf{R}^{n})^{m+1}$. *T* stands for an *m*-linear singular integral operator defined by

$$T(f_{1},\ldots,f_{m}) (x)= \int_{\mathbf{R}^{n}}\cdots \int_{\mathbf{R}^{n}}K(x,y_{1},y_{2},\ldots,y_{m})\prod_{j=1}^{m}f _{j}(y_{j})\,dy_{1}\cdots \,dy_{m}, $$

where $f_{j}$ ($j=1,\ldots,m$) are smooth functions with compact support, and $x\notin \bigcap_{j=1}^{m} \operatorname{supp} f_{j}$.

If the kernel *K* satisfies the following two conditions:

1. For some $C>0$ and all $(y_{0},y_{1},y_{2},\ldots,y_{m})\in ( \mathbf{R}^{n})^{m+1}$ defined away from the diagonal,
  1 $$ \bigl\vert K(y_{0},y_{1},y_{2}, \ldots,y_{m})\bigr\vert \leq \frac{C}{(\sum_{k,l=0}^{m} \vert y_{k}-y_{l}\vert )^{mn}}; $$
2. Whenever $i=1,\ldots,m$, $C_{k_{i}}$ are positive constants for any $(k_{1},k_{2},\ldots,k_{m})\in \mathcal{N}_{+}$,
  2 $$\begin{aligned}& \biggl( \int_{2^{k_{m}}\vert y_{0}-y_{0}^{\prime} \vert \leq \vert y_{m}-y_{0}\vert < 2^{k_{m}+1} \vert y_{0}-y_{0}^{\prime} \vert }\cdots \int_{2^{k_{1}}\vert y_{0}-y_{0}^{\prime} \vert \leq \vert y_{1}-y_{0}\vert < 2^{k_{1}+1} \vert y_{0}-y_{0}^{\prime} \vert }\bigl\vert K(y_{0},y_{1},\ldots,y_{m}) \\& \qquad {}-K \bigl(y_{0}^{\prime},y_{1},\ldots,y_{m} \bigr)\bigr\vert ^{q}\,dy_{1} \cdots \,dy_{m} \biggr)^{\frac{1}{q}} \\& \quad \leq C\bigl\vert y_{0}-y_{0}^{\prime}\bigr\vert ^{-\frac{mn}{q^{\prime}}}\prod_{i=1}^{m}C_{k_{i}}2^{-\frac{n}{q ^{\prime}}k_{i}}, \end{aligned}$$

where $(q,q^{\prime})$ is a fixed pair of positive numbers satisfying $\frac{1}{q}+\frac{1}{q^{\prime}}=1$ and $1< q<\infty $, then we call *T* an *m*-linear singular integral operator with generalized kernel.

If the kernel *K* satisfies condition (1) and the following condition:

3 $$\begin{aligned}& \bigl\vert K(y_{0},\ldots, y_{j},\ldots,y_{m})-K\bigl(y_{0},\ldots, y^{\prime }_{j},\ldots,y_{m}\bigr)\bigr\vert \\& \quad \leq \frac{C\vert y_{j}-y^{\prime }_{j}\vert ^{\varepsilon }}{(\sum_{k,l=0}^{m}\vert y_{k}-y_{l}\vert )^{mn+\varepsilon }}, \end{aligned}$$

for some $\varepsilon >0$, $\vert y_{j}-y^{\prime }_{j}\vert \leq \frac{1}{2} \max_{0\leq k\leq m}\vert y_{j}-y_{k}\vert $ whenever $0\leq j\leq m$, then we call *T* a standard *m*-linear Calderón-Zygmund kernel.

If $K(y_{0},y_{1},\ldots, y_{m})$ is defined away from the diagonal $y_{0}=y_{1}=\cdots =y_{m}$ in $(\mathbf{R}^{n})^{m+1}$, then it is called an *m*-linear Calderón-Zygmund kernel of type *κ* when it satisfies condition (1) and the following condition:

4 $$\begin{aligned}& \bigl\vert K(y_{0},\ldots, y_{j},\ldots,y_{m})-K\bigl(y_{0},\ldots, y'_{j},\ldots,y_{m}\bigr)\bigr\vert \\& \quad \leq \frac{C}{(\vert y_{0}-y_{1}\vert +\cdots +\vert y_{0}-y_{m}\vert )^{mn}}\kappa \biggl( \frac{\vert y_{j}-y'_{j}\vert }{\vert y_{0}-y_{1}\vert +\cdots +\vert y_{0}-y_{m}\vert } \biggr), \end{aligned}$$

where $\kappa (t)$ is a non-negative and non-decreasing function on $\mathbf{R}^{+}$ and $\vert y_{j}-y'_{j}\vert \leq \frac{1}{2} \max_{1\leq k \leq m}\vert y_{0}-y_{k}\vert $ whenever $0\leq j\leq m$.

It is obvious that condition (4) becomes condition (3) when $\kappa (t)=t^{\varepsilon }$ for some $\varepsilon >0$ and condition (4) implies condition (2) by putting $C_{k_{i}}=\kappa (2^{-k_{i}})^{ \frac{1}{m}}$, $i=1,\ldots,m$, and any $1< q<\infty $. Therefore, the standard *m*-linear Calderón-Zygmund kernel is a special case of the *m*-linear Calderón-Zygmund kernel of type *κ*. And the multilinear singular integral with the kernel of type *κ* can be taken as a special situation of the multilinear singular integral operator with generalized kernel defined in Definition 1.1. These facts illustrate that our results obtained in this paper will improve most of the earlier conclusions by weakening the conditions of the kernel.

### Definition 1.2

Let *T* be an *m*-linear singular integral operator with generalized kernel, $\vec{b}=(b_{1},\ldots,b_{m}) \in BMO^{m}$ is a group of locally integrable functions and $\vec{f}=(f_{1},\ldots,f_{m})$. Then the *m*-linear iterated commutator generated by *T* and *b⃗* is defined to be

$$T_{\Pi \vec{b}}(f_{1},\ldots,f_{m})= \bigl[b_{1},\bigl[b_{2},\ldots, \bigl[b_{m-1},[b _{m},T]_{m}\bigr]_{m-1}\cdots \bigr]_{2} \bigr]_{1}(\vec{f}). $$

If *T* is connected in the usual way to the kernel *K* studied in this paper, then we can write

$$\begin{aligned}& T_{\Pi \vec{b}}(f_{1},\ldots,f_{m}) (x) \\& \quad = \int_{(\mathbf{R}^{n})^{m}}\prod_{j=1}^{m} \bigl(b_{j}(x)-b_{j}(y_{j})\bigr)K(x,y _{1},\ldots,y_{m})f_{1}(y_{1})\cdots f_{m}(y_{m})\,dy_{1}\cdots \,dy_{m}. \end{aligned}$$

We also denote

$$T_{b_{j}}^{j}(\vec{f}) (x)=b_{j}(x)T(f_{1}, \ldots,f_{m}) (x)-T(f_{1}, \ldots,f_{j-1},b_{j}f_{j},f_{j+1}, \ldots,f_{m}) (x). $$

### Definition 1.3

Take positive integers *j* and *m* satisfying $1\leq j\leq m$, and let $C^{m}_{j}$ be a family of all finite subsets $\sigma =\{\sigma (1),\ldots,\sigma (j)\}$ of $\{1,\ldots,m\}$ with *j* different elements. If $k< l$, then $\sigma (k)<\sigma (l)$. For any $\sigma \in C^{m}_{j}$, let $\sigma^{\prime}=\{1,\ldots,m\} \backslash \sigma $ be the complementary sequence. In particular, $C^{m}_{0}=\emptyset $. For an *m*-tuple *b⃗* and $\sigma \in C^{m}_{j}$, the *j*-tuple $\vec{b}_{\sigma }=(b_{\sigma (1)},\ldots,b_{\sigma (j)})$ is a finite subset of $\vec{b}=(b_{1},\ldots,b_{m})$.

Let *T* be an *m*-linear singular integral operator with generalized kernel, $\sigma \in C^{m}_{j}$, $\vec{b}_{\sigma }=(b_{\sigma (1)}, \ldots,b_{\sigma (j)}) \in BMO^{j}$, the iterated commutator is given by

$$T_{\Pi \vec{b}_{\sigma }}(f_{1},\ldots,f_{m})= \bigl[b_{\sigma (1)},\bigl[b_{ \sigma (2)},\ldots, \bigl[b_{\sigma (j-1)},[b_{\sigma (j)},T]_{\sigma (j)} \bigr]_{ \sigma (j-1)}\cdots \bigr]_{\sigma (2)}\bigr]_{\sigma (1)}( \vec{f}). $$

It can also be written as

$$\begin{aligned}& T_{\Pi \vec{b}_{\sigma }}(f_{1},\ldots,f_{m}) (x) \\& \quad = \int_{(\mathbf{R}^{n})^{m}}\prod_{i=1}^{j} \bigl(b_{\sigma (i)}(x)-b _{\sigma (i)}(y_{\sigma (i)}) \bigr)K(x,y_{1},\ldots,y_{m})f_{1}(y_{1}) \cdots f_{m}(y_{m})\,d\vec{y}, \end{aligned}$$

where $d\vec{y}=dy_{1}\cdots dy_{m}$. Obviously, $T_{\Pi \vec{b}_{ \sigma }}=T_{\Pi \vec{b}}$ when $\sigma =\{1,2,\ldots,m\}$, and $T_{\Pi \vec{b}_{\sigma }}=T_{b_{j}}^{j}$ when $\sigma =\{j\}$.

### Definition 1.4

The Hardy-Littlewood maximal operator *M* is given by

$$M(f) (x)=\sup_{B\ni x}\frac{1}{\vert B\vert } \int_{B}\bigl\vert f(y)\bigr\vert \,dy, $$

where the supremum is taken over all balls which contain *x*. We define the operator $M_{s}(f)$ by $M_{s}(f)=M(\vert f\vert ^{s})^{1/s}$, $0< s<\infty $.

The sharp maximal operator $M^{\sharp }$ is defined by

$$M^{\sharp }(f) (x)=\sup_{B\ni x}\frac{1}{\vert B\vert } \int_{B}\bigl\vert f(y)-f_{B}\bigr\vert \,dy \sim \sup_{B\ni x}\inf_{a\in \mathbf{C}}\frac{1}{\vert B\vert } \int_{B}\bigl\vert f(y)-a\bigr\vert \,dy. $$

Denote the *l*-sharp maximal operator by $M_{l}^{\sharp }(f)=M^{ \sharp }(\vert f\vert ^{l})^{1/l}$, $0< l<1$.

### Definition 1.5

([8])

Let *ω* be a non-negative measurable function. For $1< p<\infty $, $\omega \in A_{p}$ if there exists a positive constant *C* independent of every *Q* in $\mathbf{R}^{n}$ such that

$$\biggl(\frac{1}{\vert Q\vert } \int_{Q} \omega (x)\,dx \biggr) \biggl(\frac{1}{\vert Q\vert } \int_{Q} \omega (x)^{1-p'}\,dx \biggr)^{p-1} \leq C, $$

where *Q* represents a cube with the side parallel to the coordinate axes, $1/p+1/p'=1$. When $p=1$, *ω* belongs to $A_{1}$, if there exists a constant $C>0$, and for any cube *Q* such that

$$\frac{1}{\vert Q\vert } \int_{Q} \omega (y)\,dy\leq C\omega (x),\quad \mbox{a.e. }x \in Q. $$

### Definition 1.6

Let $p(\cdot):\mathbf{R}^{n}\rightarrow [1,\infty)$ be a measurable function. Define the variable exponent Lebesgue space $L^{p(\cdot)}(\mathbf{R}^{n})$ by

$$L^{p(\cdot)}\bigl(\mathbf{R}^{n}\bigr)= \biggl\{ f \mbox{ measurable}: \int_{\mathbf{R}^{n}} \biggl( \frac{\vert f(x)\vert }{\lambda } \biggr) ^{p(x)}\,dx< \infty \mbox{ for some constant } \lambda >0 \biggr\} . $$

By associating with the norm

$$\Vert f\Vert _{L^{p(\cdot)}(\mathbf{R}^{n})}=\inf \biggl\{ \lambda >0: \int_{\mathbf{R}^{n}} \biggl( \frac{\vert f(x)\vert }{\lambda } \biggr) ^{p(x)}\,dx \leq 1 \biggr\} , $$

the set $L^{p(\cdot)}(\mathbf{R}^{n})$ comes to be a Banach space.

### Definition 1.7

Let $\mathcal{P}(\mathbf{R}^{n})$ represent the set of measurable functions $p(\cdot):\mathbf{R}^{n}\rightarrow [1,\infty)$ satisfying

$$1< p_{-}:=\mathop{\operatorname{essinf}} _{x\in \mathbf{R}^{n}}p(x) \quad \mbox{and}\quad p_{+}:= \mathop{\operatorname{esssup}} _{x\in \mathbf{R}^{n}}p(x)< \infty, $$

and $\mathcal{B}(\mathbf{R}^{n})$ represent the set of all $p(\cdot) \in \mathcal{P}(\mathbf{R}^{n})$ for which the Hardy-Littlewood maximal operator *M* is bounded on $L^{p(\cdot)}(\mathbf{R}^{n})$.

## Main results

In this part, we will give the main results in this paper.

### Theorem 2.1

*Let* $m\geq 2$, *T* *be an* *m*-*linear singular integral operator with generalized kernel defined by Definition* 1.1 *and* $\sum_{k_{i}=1}^{\infty }k_{i}C_{k_{i}}<\infty $, $i=1,\ldots,m$. *Assume for fixed* $1\leq r_{1},\ldots,r_{m}\leq q^{\prime }$ *with* $1/r=1/r_{1}+\cdots +1/r_{m}$ *that* *T* *is bounded from* $L^{r_{1}} \times \cdots \times L^{r_{m}}$ *into* $L^{r,\infty }$. *If* $\vec{b} \in BMO^{m}$, $0<\delta <\frac{1}{m}$, $\delta <\varepsilon <\infty $ *and* $q^{\prime }< s<\infty $, *then there is a constant* $C>0$ *such that*

$$\begin{aligned} M^{\sharp }_{\delta }\bigl(T_{\Pi \vec{b}}(\vec{f})\bigr) (x) \leq & C\prod_{j=1} ^{m}\Vert b_{j} \Vert _{BMO} \Biggl( \prod_{k=1}^{m}M_{s}(f_{k}) (x)+M_{\varepsilon }\bigl(T(\vec{f})\bigr) (x) \Biggr) \\ &{} +C\sum_{j=1}^{m-1} \sum _{\sigma \in C_{j}^{m}}\prod_{i=1}^{j} \Vert b_{\sigma (i)}\Vert _{BMO} M_{\varepsilon } \bigl(T_{\Pi \vec{b}_{\sigma \prime }}(\vec{f}) (x)\bigr) \end{aligned}$$

*for all m*-*tuples* $\vec{f}=(f_{1},\ldots,f_{m})$ *of bounded measurable functions with compact support*.

### Theorem 2.2

*Let* $m\geq 2$, *T* *be an* *m*-*linear singular integral operator with generalized kernel defined by Definition* 1.1 *and* $\sum_{k_{i}=1}^{\infty }k_{i}C_{k_{i}}<\infty $, $i=1,\ldots,m$. *Assume for fixed* $1\leq r_{1},\ldots,r_{m}\leq q^{\prime }$ *with* $1/r=1/r_{1}+\cdots +1/r_{m}$ *that* *T* *is bounded from* $L^{r_{1}} \times \cdots \times L^{r_{m}}$ *into* $L^{r,\infty }$. *If* $\vec{b} \in BMO^{m}$, *then for any* $q'< p_{1},\ldots,p_{m}<\infty $ *with* $1/p=1/p_{1}+\cdots +1/p_{m}$, $T_{\Pi \vec{b}}$ *is bounded from* $L^{p_{1}}(w_{1})\times \cdots \times L^{p_{m}}(w_{m})$ *into* $L^{p}(w)$, *where* $(w_{1},\ldots,w_{m})\in (A_{p_{1}/q'},\ldots,A _{p_{m}/q'})$ *and* $w=\prod_{j=1}^{m}w_{j}^{\frac{p}{p_{j}}}$.

### Theorem 2.3

*Let* $m\geq 2$, $p(\cdot),p_{1}(\cdot), \ldots,p_{m}(\cdot)\in \mathcal{B}(\mathbf{R}^{n})$ *with* $1/p( \cdot)=1/p_{1}(\cdot)+\cdots +1/p_{m}(\cdot)$. *Let* $q^{j}_{0}$ *be given as in Lemma* 3.8 *for* $p_{j}(\cdot)$, $j=1,\ldots,m$. *T* *is an* *m*-*linear singular integral operator with generalized kernel defined by Definition* 1.1, $1< q'<\min_{1\leq j\leq m}q_{0}^{j}$ *and* $\sum_{k_{i}=1}^{\infty }k_{i}C_{k_{i}}<\infty $, $i=1,\ldots,m$. *Assume for fixed* $1\leq r_{1},\ldots,r_{m}\leq q^{\prime }$ *with* $1/r=1/r_{1}+\cdots +1/r_{m}$ *that* *T* *is bounded from* $L^{r_{1}} \times \cdots \times L^{r_{m}}$ *into* $L^{r,\infty }$. *If* $\vec{b} \in BMO^{m}$, *then* $T_{\Pi \vec{b}}$ *is bounded from* $L^{p_{1}( \cdot)}(\mathbf{R}^{n})\times \cdots \times L^{p_{m}(\cdot)}( \mathbf{R}^{n})$ *into* $L^{p(\cdot)}(\mathbf{R}^{n})$.

## Preliminaries

Next, we give some requisite lemmas.

### Lemma 3.1

([9, 10])

*Suppose* $0< p< q<\infty $, *then there exists a constant* $C=C_{p,q}>0$ *such that for any measurable function* *f*,

$$\vert Q\vert ^{-1/p} \Vert f\Vert _{L^{p}(Q)} \leq C \vert Q\vert ^{-1/q} \Vert f\Vert _{L^{q,\infty }(Q)} . $$

### Lemma 3.2

([11])

*Let* $f \in BMO(\mathbf{R}^{n})$, $1\leq p<\infty $, $r_{1}>0$, $r_{2}>0$ *and* $x\in \mathbf{R}^{n} $. *Then*

$$\biggl( \frac{1}{\vert B(x,r_{1})\vert } \int_{B(x,r_{1})} \bigl\vert f(y)-f_{B(x,r_{2})}\bigr\vert ^{p}\,dy \biggr) ^{1/p} \leq C \biggl( 1+ \biggl\vert \ln \frac{r_{1}}{r_{2}}\biggr\vert \biggr) \Vert f\Vert _{BMO}, $$

*where* *C* *is a positive constant independent of* *f*, *x*, $r_{1}$ *and* $r_{2}$.

### Lemma 3.3

([10, 12])

*Suppose* $0< p,\delta < \infty $ *and* $w\in A_{\infty }$. *There is a positive constant* *C* *depending on the* $A_{\infty }$ *constant of* *w* *such that*

$$\int_{\mathbf{R}^{n}}\bigl[ M_{\delta }(f) (x)\bigr]^{p}w(x)\,dx \leq C \int_{\mathbf{R}^{n}} \bigl[ M^{\sharp }_{\delta }(f) (x) \bigr]^{p}w(x)\,dx $$

*for every function* *f* *such that the left*-*hand side is finite*.

### Lemma 3.4

([13])

*Let* $(w_{1},\ldots,w_{m}) \in (A_{p_{1}},\ldots,A_{p_{m}})$ *with* $1\leq p_{1},\ldots,p_{m}< \infty $, *and* $0<\theta_{1},\ldots,\theta_{m}<1$ *satisfying* $\theta_{1}+\cdots +\theta_{m}=1$, *then* $w_{1}^{\theta_{1}}\cdots w _{m}^{\theta_{m}} \in A_{\max \{p_{1},\ldots,p_{m}\}} $.

### Lemma 3.5

([6])

*Let* $m\geq 2$, *T* *be an* *m*-*linear singular integral operator with generalized kernel defined by Definition* 1.1 *and* $\sum_{k_{i}=1}^{\infty }C_{k_{i}}<\infty $, $i=1,\ldots,m$. *Suppose for fixed* $1\leq r_{1},\ldots,r_{m}\leq q ^{\prime }$ *with* $1/r=1/r_{1}+\cdots +1/r_{m}$ *that* *T* *is bounded from* $L^{r_{1}}\times \cdots \times L^{r_{m}}$ *into* $L^{r,\infty }$. *If* $0<\delta <1/m $, *then*

$$M^{\sharp }_{\delta }\bigl(T(\vec{f})\bigr) (x)\leq C\prod _{j=1}^{m}M_{q'}(f_{j}) (x) $$

*for all* *m*-*tuples* $\vec{f}=(f_{1},\ldots,f_{m})$ *of bounded measurable functions with compact support*.

### Lemma 3.6

([8])

*For* $\omega \in A_{p}$, $1< p<\infty $, *there are* $\omega \in A_{r}$ *for all* $r>p$ *and* $\omega \in A_{q}$ *for some* $1< q< p$.

### Lemma 3.7

([6])

*Let* $m\geq 2$, *T* *be an* *m*-*linear singular integral operator with generalized kernel defined by Definition* 1.1 *and* $\sum_{k_{i}=1}^{\infty }k_{i}C_{k_{i}}<\infty $, $i=1,\ldots,m$. *Suppose for fixed* $1\leq r_{1},\ldots,r_{m} \leq q^{\prime }$ *with* $1/r=1/r_{1}+\cdots +1/r_{m}$ *that* *T* *is bounded from* $L^{r_{1}}\times \cdots \times L^{r_{m}}$ *into* $L^{r,\infty }$. *If* $b_{j}\in BMO$ *for* $j=1,\ldots,m$, $0<\delta <\frac{1}{m}$, $\delta <\varepsilon <\infty $ *and* $q^{\prime }< s<\infty $, *then*

$$M^{\sharp }_{\delta }\bigl(T_{b_{j}}^{j}(\vec{f}) \bigr) (x)\leq C \Vert b_{j}\Vert _{BMO} \Biggl( M_{\varepsilon }\bigl(T(\vec{f})\bigr) (x) +\prod_{i=1}^{m} M_{s}(f_{i}) (x) \Biggr) $$

*for all* *m*-*tuples* $\vec{f}=(f_{1},\ldots,f_{m})$ *of bounded measurable functions with compact support*.

The above result can be obtained from the proof of Theorem 2.2 in [6].

### Lemma 3.8

([14])

*Suppose* $p(\cdot)\in \mathcal{P}(\mathbf{R}^{n})$. *M* *is bounded on* $L^{p(\cdot)}( \mathbf{R}^{n})$ *if and only if for some* $1< q_{0}<\infty $, $M_{q_{0}}$ *is bounded on* $L^{p(\cdot)}(\mathbf{R}^{n})$, *where* $M_{q_{0}}(f)=[M(\vert f\vert ^{q_{0}})]^{1/q_{0}} $.

### Lemma 3.9

([3])

*Let* $p(\cdot), p_{1}( \cdot), \ldots, p_{m}(\cdot)\in \mathcal{P}(\mathbf{R}^{n})$ *satisfying* $1/p(x)=1/p_{1}(x)+\cdots +1/p_{m}(x)$. *For any* $f_{j} \in L^{p_{j}(\cdot)}(\mathbf{R}^{n})$, $j=1,\ldots, m$, *there is*

$$\Biggl\Vert \prod_{j=1}^{m}f_{j} \Biggr\Vert _{L^{p(\cdot)}(\mathbf{R}^{n}) } \leq 2^{m-1}\prod _{j=1}^{m}\Vert f_{j}\Vert _{L^{p_{j}(\cdot)}(\mathbf{R} ^{n})}. $$

### Lemma 3.10

([15])

*Given a family of ordered pairs of measurable functions* $\mathcal{F}$, *for some fixed* $0< p_{0}<\infty $, *any* $(f,g)\in \mathcal{F}$ *and any* $w\in A_{1}$,

$$\int_{\mathbf{R}^{n}}\bigl\vert f(x)\bigr\vert ^{p_{0}}w(x)\,dx \leq C_{0} \int_{\mathbf{R} ^{n}}\bigl\vert g(x)\bigr\vert ^{p_{0}}w(x)\,dx. $$

*Suppose* $p(\cdot)\in \mathcal{P}(\mathbf{R}^{n})$ *satisfying* $p_{0}\leq p_{-}$. *If* $(\frac{p(\cdot)}{p_{0}})'\in \mathcal{B}( \mathbf{R}^{n})$, *then there exists a constant* $C>0$ *such that for any* $(f,g)\in \mathcal{F}$, $\Vert f\Vert _{L^{p(\cdot)}(\mathbf{R}^{n})}\leq C \Vert g\Vert _{L^{p(\cdot)}(\mathbf{R}^{n})}$.

### Lemma 3.11

([14])

*If* $p(\cdot)\in \mathcal{P}( \mathbf{R}^{n})$, *then the following four conditions are equivalent*.

1. $p(\cdot)\in \mathcal{B}(\mathbf{R}^{n})$.
2. $p'(\cdot)\in \mathcal{B}(\mathbf{R}^{n})$.
3. *For some* $1< p_{0}< p_{-}$, $\frac{p(\cdot)}{p_{0}}\in \mathcal{B}(\mathbf{R}^{n})$.
4. *For some* $1< p_{0}< p_{-}$, $(\frac{p(\cdot)}{p_{0}})'\in \mathcal{B}(\mathbf{R}^{n})$.

### Lemma 3.12

([15])

*For* $p(\cdot)\in \mathcal{P}(\mathbf{R}^{n})$, $C_{0}^{\infty }(\mathbf{R}^{n})$ *is dense in* $L^{p(\cdot)}(\mathbf{R}^{n})$.

## Proof of the main results

We will give the proof of the three theorems in the following article.

### Proof of Theorem 2.1

For the sake of simplicity, we only consider the case $m=2$. The proof of other cases is similar.

Let $f_{1}$, $f_{2}$ be bounded measurable functions with compact support, $b_{1}, b_{2}\in BMO$. Then, for any constant $\lambda_{1}$ and $\lambda_{2}$,

$$\begin{aligned} T_{\Pi \vec{b}}(\vec{f}) (x) =&\bigl(b_{1}(x)- \lambda_{1}\bigr) \bigl(b_{2}(x)-\lambda _{2} \bigr)T(f_{1},f_{2}) (x)-\bigl(b_{1}(x)- \lambda_{1}\bigr) \\ &{}\times T\bigl(f_{1},(b_{2}-\lambda_{2})f_{2} \bigr) (x)-\bigl(b_{2}(x)-\lambda_{2}\bigr)T\bigl((b _{1}-\lambda_{1})f_{1},f_{2}\bigr) (x) \\ &{}+T\bigl((b_{1}-\lambda_{1})f_{1},(b_{2}- \lambda_{2})f_{2}\bigr) (x) \\ =&-\bigl(b_{1}(x)-\lambda_{1}\bigr) \bigl(b_{2}(x)- \lambda_{2}\bigr)T(f_{1},f_{2}) (x)+\bigl(b _{1}(x)-\lambda_{1}\bigr) \\ &{}\times T_{b_{2}-\lambda_{2}}^{2}(f_{1},f_{2}) (x)+\bigl(b_{2}(x)-\lambda _{2}\bigr)T_{b_{1}-\lambda_{1}}^{1}(f_{1},f_{2}) (x) \\ &{}+T\bigl((b_{1}-\lambda_{1})f_{1},(b_{2}- \lambda_{2})f_{2}\bigr) (x). \end{aligned}$$

Let $C_{0}$ be a constant determined later. Fixed $x \in \mathbf{R} ^{n} $, for any ball $B(x_{0},r_{B})$ containing *x* and $0<\delta < \frac{1}{2}$, we have

$$\begin{aligned}& \biggl( \frac{1}{\vert B\vert } \int_{B} \bigl\vert \bigl\vert T_{\Pi \vec{b}}(\vec{f}) (z)\bigr\vert ^{ \delta }-\vert C_{0}\vert ^{\delta } \bigr\vert \,dz \biggr) ^{\frac{1}{\delta }} \\& \quad \leq \biggl( \frac{1}{\vert B\vert } \int_{B} \bigl\vert T_{\Pi \vec{b}}(\vec{f}) (z)-C_{0}\bigr\vert ^{ \delta }\,dz \biggr) ^{\frac{1}{\delta }} \\& \quad \leq C \biggl( \frac{1}{\vert B\vert } \int_{B} \bigl\vert \bigl(b_{1}(z)- \lambda_{1}\bigr) \bigl(b_{2}(z)-\lambda_{2} \bigr)T(f_{1},f_{2}) (z)\bigr\vert ^{\delta }\,dz \biggr) ^{\frac{1}{\delta }} \\& \qquad {} +C \biggl( \frac{1}{\vert B\vert } \int_{B} \bigl\vert \bigl(b_{1}(z)- \lambda_{1}\bigr)T_{b_{2}-\lambda_{2}}^{2}(f_{1},f_{2}) (z)\bigr\vert ^{\delta }\,dz \biggr) ^{\frac{1}{ \delta }} \\& \qquad {}+C \biggl( \frac{1}{\vert B\vert } \int_{B} \bigl\vert \bigl(b_{2}(z)- \lambda_{2}\bigr)T_{b_{1}-\lambda_{1}}^{1}(f_{1},f_{2}) (z)\bigr\vert ^{\delta }\,dz \biggr) ^{\frac{1}{ \delta }} \\& \qquad {} +C \biggl( \frac{1}{\vert B\vert } \int_{B} \bigl\vert T\bigl((b_{1}- \lambda_{1})f_{1},(b_{2}-\lambda_{2})f_{2} \bigr) (z)-C_{0}\bigr\vert ^{\delta }\,dz \biggr) ^{\frac{1}{ \delta }} \\& \quad :=I+II+III+IV. \end{aligned}$$

Then we analyze each part separately.

Let $B^{*}=16 B $ and $\lambda_{j}=(b_{j})_{B^{*}}= \frac{1}{\vert 16B\vert } \int_{16B}b_{j}(x)\,dx $, $j=1,2$. Since $0<\delta <\frac{1}{2}$ and $0<\delta <\varepsilon <\infty $, there exists *l* such that $1< l<\min \{\frac{\varepsilon }{\delta },\frac{1}{1-\delta }\}$. Then $l\delta <\varepsilon $ and $l'\delta >1$. Choose $q_{1},q_{2}\in (1, \infty)$ such that $\frac{1}{q_{1}}+\frac{1}{q_{2}}=\frac{1}{l'}$, then $\frac{1}{q_{1}}+\frac{1}{q_{2}}+\frac{1}{l}=1$ and $q_{1}\delta >1$, $q_{2} \delta >1$. By Hölder’s inequality and Lemma 3.2, we have

$$\begin{aligned} I \leq & C \biggl( \frac{1}{\vert B\vert } \int_{B} \bigl\vert b_{1}(z)- \lambda_{1}\bigr\vert ^{\delta }\bigl\vert b_{2}(z)-\lambda_{2}\bigr\vert ^{\delta }\bigl\vert T(f_{1},f_{2}) (z)\bigr\vert ^{\delta }\,dz \biggr) ^{\frac{1}{\delta }} \\ \leq & C \biggl( \frac{1}{\vert B\vert } \int_{B} \bigl\vert b_{1}(z)- \lambda_{1}\bigr\vert ^{\delta q _{1}}\,dz \biggr) ^{\frac{1}{\delta q_{1}}} \biggl( \frac{1}{\vert B\vert } \int_{B} \bigl\vert b_{2}(z)- \lambda_{2}\bigr\vert ^{\delta q_{2}}\,dz \biggr) ^{\frac{1}{\delta q _{2}}} \\ &{}\times \biggl( \frac{1}{\vert B\vert } \int_{B} \bigl\vert T(f_{1},f_{2}) (z)\bigr\vert ^{\delta l}\,dz \biggr) ^{\frac{1}{\delta l}} \\ \leq & C \Vert b_{1}\Vert _{BMO} \Vert b_{2}\Vert _{BMO} M_{\delta l}\bigl(T(f_{1},f_{2}) \bigr) (x) \\ \leq &C \Vert b_{1}\Vert _{BMO} \Vert b_{2}\Vert _{BMO} M_{\varepsilon }\bigl(T(f_{1},f _{2})\bigr) (x). \end{aligned}$$

It follows from Hölder’s inequality that

$$\begin{aligned} \mathit{II} \leq & C \biggl( \frac{1}{\vert B\vert } \int_{B} \bigl\vert b_{1}(z)- \lambda_{1}\bigr\vert ^{ \delta l'}\,dz \biggr) ^{\frac{1}{\delta l'}} \biggl( \frac{1}{\vert B\vert } \int _{B} \bigl\vert T_{b_{2}-\lambda_{2}}^{2}(f_{1},f_{2}) (z)\bigr\vert ^{\delta l}\,dz \biggr) ^{\frac{1}{\delta l}} \\ \leq & C \Vert b_{1}\Vert _{BMO} M_{\delta l} \bigl(T_{b_{2}-\lambda_{2}}^{2}(f _{1},f_{2})\bigr) (x) \\ \leq & C \Vert b_{1}\Vert _{BMO} M_{\varepsilon } \bigl(T_{b_{2}-\lambda_{2}}^{2}(f _{1},f_{2})\bigr) (x) \\ =& C \Vert b_{1}\Vert _{BMO} M_{\varepsilon } \bigl(T_{b_{2}}^{2}(f_{1},f_{2})\bigr) (x). \end{aligned}$$

Similarly,

$$\mathit{III}\leq C \Vert b_{2}\Vert _{BMO} M_{\varepsilon } \bigl(T_{b_{1}}^{1}(f_{1},f_{2})\bigr) (x). $$

In the next, we analyze *IV*. We split $f_{i}$ into two parts, $f_{i}=f_{i}^{0}+f_{i}^{\infty }$, where $f_{i}^{0}=f_{\chi_{B^{*}}}$ and $f_{i}^{\infty }=f_{i}-f_{i}^{0}$, $i=1,2$. Choose $z_{0} \in 3B \backslash 2B$ and $C_{0}=T((b_{1}-\lambda_{1})f_{1}^{\infty },(b_{2}- \lambda_{2})f_{2}^{\infty })(z_{0})$, then

$$\begin{aligned} \mathit{IV} \leq & C \biggl( \frac{1}{\vert B\vert } \int_{B} \bigl\vert T\bigl((b_{1}- \lambda_{1})f_{1}^{0},(b_{2}- \lambda_{2})f_{2}^{0}\bigr) (z)\bigr\vert ^{\delta }\,dz \biggr) ^{\frac{1}{ \delta }} \\ & {}+C \biggl( \frac{1}{\vert B\vert } \int_{B} \bigl\vert T\bigl((b_{1}- \lambda_{1})f_{1}^{0},(b_{2}- \lambda_{2})f_{2}^{\infty }\bigr) (z)\bigr\vert ^{\delta }\,dz \biggr) ^{\frac{1}{ \delta }} \\ &{} + C \biggl( \frac{1}{\vert B\vert } \int_{B} \bigl\vert T\bigl((b_{1}- \lambda_{1})f_{1}^{\infty},(b_{2}- \lambda_{2})f_{2}^{0}\bigr) (z)\bigr\vert ^{\delta }\,dz \biggr) ^{\frac{1}{ \delta }} \\ &{} +C \biggl( \frac{1}{\vert B\vert } \int_{B} \bigl\vert T\bigl((b_{1}- \lambda_{1})f_{1}^{\infty},(b_{2}- \lambda_{2})f_{2}^{\infty }\bigr) (z)-C_{0} \bigr\vert ^{\delta }\,dz \biggr) ^{\frac{1}{\delta }} \\ :=& IV_{1}+IV_{2}+IV_{3}+IV_{4}. \end{aligned}$$

Then we estimate each part respectively.

It follows from $0<\delta <r<\infty $ and Lemma 3.1 that

$$\begin{aligned} IV_{1} \leq & C \vert B\vert ^{-\frac{1}{\delta }} \bigl\Vert T \bigl((b_{1}-\lambda_{1})f_{1}^{0},(b_{2}- \lambda_{2})f_{2}^{0}\bigr)\bigr\Vert _{ L^{\delta }(B)} \\ \leq & C \vert B\vert ^{-\frac{1}{r}} \bigl\Vert T\bigl((b_{1}- \lambda_{1})f_{1}^{0},(b_{2}- \lambda_{2})f_{2}^{0}\bigr)\bigr\Vert _{ L^{r,\infty }(B)}. \end{aligned}$$

Notice that *T* is bounded from $L^{r_{1}}\times L^{r_{2}}$ into $L^{r,\infty }$, $1\leq r_{1},r_{2}\leq q^{\prime }$ with $1/r=1/r _{1}+1/r_{2}$. Let $t=\frac{s}{q^{\prime }}$. Since $q^{\prime }< s< \infty $, then $1< t<\infty $ and $r_{i}t\leq s$, $i=1,2$. Thus,

$$\begin{aligned} IV_{1} \leq & C \biggl( \frac{1}{\vert 16B\vert } \int_{16B} \bigl\vert b_{1}(y_{1})- \lambda_{1}\bigr\vert ^{r_{1}} \bigl\vert f_{1}(y_{1})\bigr\vert ^{r_{1}}\,dy_{1} \biggr) ^{\frac{1}{r_{1}}} \\ & {}\times \biggl( \frac{1}{\vert 16B\vert } \int_{16B} \bigl\vert b_{2}(y_{2})- \lambda_{2}\bigr\vert ^{r _{2}} \bigl\vert f_{2}(y_{2})\bigr\vert ^{r_{2}}\,dy_{2} \biggr) ^{\frac{1}{r_{2}}} \\ \leq & C \biggl( \frac{1}{\vert 16B\vert } \int_{16B} \bigl\vert b_{1}(y_{1})- \lambda_{1}\bigr\vert ^{r _{1}t'}\,dy_{1} \biggr) ^{\frac{1}{r_{1}t'} } \biggl( \frac{1}{\vert 16B\vert } \int_{16B} \bigl\vert f_{1}(y_{1})\bigr\vert ^{r_{1}t}\,dy_{1} \biggr) ^{\frac{1}{r_{1}t}} \\ & {}\times \biggl( \frac{1}{\vert 16B\vert } \int_{16B} \bigl\vert b_{2}(y_{2})- \lambda_{2}\bigr\vert ^{r _{2}t'}\,dy_{2} \biggr) ^{\frac{1}{r_{2}t'}} \biggl( \frac{1}{\vert 16B\vert } \int_{16B} \bigl\vert f_{2}(y_{2})\bigr\vert ^{r_{2}t}\,dy_{2} \biggr) ^{\frac{1}{r_{2}t}} \\ \leq & C \Vert b_{1}\Vert _{BMO} \Vert b_{2}\Vert _{BMO} \biggl( \frac{1}{\vert 16B\vert } \int_{16B} \bigl\vert f_{1}(y_{1})\bigr\vert ^{s}\,dy_{1} \biggr) ^{\frac{1}{s}} \\ & {}\times \biggl( \frac{1}{\vert 16B\vert } \int_{16B} \bigl\vert f_{2}(y_{2})\bigr\vert ^{s}\,dy_{2} \biggr) ^{\frac{1}{s}} \\ \leq & C \Vert b_{1}\Vert _{BMO} \Vert b_{2}\Vert _{BMO} M_{s}(f_{1}) (x) M_{s}(f_{2}) (x). \end{aligned}$$

Since $y_{1}\in 16B$, $y_{2}\in (16B)^{c}$, $x_{0}\in B$ and $z\in B$, then by Lemma 3.2,

$$\begin{aligned} IV_{2} \leq & C \biggl( \frac{1}{\vert B\vert } \int_{B} \biggl( \int_{(16B)^{c}} \int_{16B} \bigl\vert K(z,y_{1},y_{2}) \bigr\vert \bigl\vert b_{1}(y_{1})-\lambda_{1} \bigr\vert \bigl\vert f_{1}(y_{1})\bigr\vert \bigl\vert b_{2}(y_{2})-\lambda_{2}\bigr\vert \\ &{}\times \bigl\vert f_{2}(y_{2})\bigr\vert \,dy_{1}\,dy_{2} \biggr)^{\delta }\,dz \biggr)^{\frac{1}{ \delta }} \\ \leq & C \biggl( \frac{1}{\vert B\vert } \int_{B} \biggl( \int_{(16B)^{c}} \biggl( \int_{16B} \bigl\vert b_{1}(y_{1})- \lambda_{1}\bigr\vert \bigl\vert f_{1}(y_{1}) \bigr\vert \,dy_{1} \biggr) \\ &{}\times \frac{\vert b_{2}(y_{2})-\lambda_{2}\vert \vert f_{2}(y_{2})\vert }{\vert z-y_{2}\vert ^{2n}}\,dy _{2} \biggr)^{\delta }\,dz \biggr)^{\frac{1}{\delta }} \\ \leq & C \biggl( \int_{16B} \bigl\vert b_{1}(y_{1})- \lambda_{1}\bigr\vert \bigl\vert f_{1}(y_{1}) \bigr\vert \,dy _{1} \biggr) \sum^{\infty }_{k=4} \int_{2^{k+1}B\backslash 2^{k}B} \frac{\vert b_{2}(y_{2})-\lambda_{2}\vert \vert f_{2}(y_{2})\vert }{\vert x_{0}-y_{2}\vert ^{2n}}\,dy_{2} \\ \leq & C \biggl( \frac{1}{\vert 16B\vert } \int_{16B} \bigl\vert b_{1}(y_{1})- \lambda_{1}\bigr\vert \bigl\vert f_{1}(y_{1}) \bigr\vert \,dy_{1} \biggr) \sum^{\infty }_{k=4} 2^{-kn} \frac{1}{\vert 2^{k+1}B\vert } \\ &{}\times \int_{2^{k+1}B}\bigl\vert b_{2}(y_{2})- \lambda_{2}\bigr\vert \bigl\vert f_{2}(y_{2}) \bigr\vert \,dy _{2} \\ \leq & C \biggl( \frac{1}{\vert 16B\vert } \int_{16B} \bigl\vert b_{1}(y_{1})- \lambda_{1}\bigr\vert ^{q}\,dy_{1} \biggr)^{\frac{1}{q}} \biggl( \frac{1}{\vert 16B\vert } \int_{16B}\bigl\vert f_{1}(y_{1})\bigr\vert ^{q'}\,dy_{1} \biggr) ^{ \frac{1}{q'}} \\ &{}\times \sum^{\infty }_{k=4} 2^{-kn} \biggl(\frac{1}{\vert 2^{k+1}B\vert } \int_{2^{k+1}B} \bigl\vert b_{2}(y_{2})- \lambda_{2}\bigr\vert ^{q}\,dy_{2} \biggr) ^{ \frac{1}{q}} \\ &{}\times \biggl(\frac{1}{\vert 2^{k+1}B\vert } \int_{2^{k+1}B} \bigl\vert f_{2}(y_{2})\bigr\vert ^{q'}\,dy_{2} \biggr) ^{\frac{1}{q'}} \\ \leq & C\Vert b_{1}\Vert _{BMO} \Vert b_{2}\Vert _{BMO} M_{q'}(f_{1}) (x) M_{q'}(f _{2}) (x) \sum^{\infty }_{k=4} 2^{-kn}k \\ \leq & C\Vert b_{1}\Vert _{BMO} \Vert b_{2}\Vert _{BMO} M_{s}(f_{1}) (x) M_{s}(f_{2}) (x). \end{aligned}$$

Similarly,

$$IV_{3}\leq C\Vert b_{1}\Vert _{BMO} \Vert b_{2}\Vert _{BMO} M_{s}(f_{1}) (x) M_{s}(f _{2}) (x). $$

Since $z_{0} \in 3B\backslash 2B$, $z\in B $, $y_{1}\in (16B)^{c}$, $y_{2}\in (16B)^{c}$, then $\vert y_{1}-z_{0}\vert \geq 2\vert z-z_{0}\vert $ and $\vert y_{2}-z_{0}\vert \geq 2\vert z-z_{0}\vert $.

$$\begin{aligned} IV_{4} \leq & C \biggl(\frac{1}{\vert B\vert } \int_{B} \biggl( \int_{(16B)^{c}} \int_{(16B)^{c}} \bigl\vert K(z,y_{1},y_{2})-K(z_{0},y_{1},y_{2}) \bigr\vert \bigl\vert b_{1}(y_{1})-\lambda_{1} \bigr\vert \\ &{}\times \bigl\vert f_{1}(y_{1})\bigr\vert \bigl\vert b_{2}(y_{2})-\lambda_{2}\bigr\vert \bigl\vert f_{2}(y_{2})\bigr\vert \,dy _{1}\,dy_{2} \biggr)^{\delta }\,dz \biggr)^{\frac{1}{\delta }} \\ \leq & C \Biggl(\frac{1}{\vert B\vert } \int_{B} \Biggl( \sum_{k_{1}=1}^{\infty } \sum_{k_{2}=1}^{\infty } \int_{2^{k_{2}}\vert z-z_{0}\vert \leq \vert y_{2}-z_{0}\vert < 2^{k_{2}+1}\vert z-z_{0}\vert } \\ &{}\times \int_{2^{k_{1}}\vert z-z_{0}\vert \leq \vert y_{1}-z_{0}\vert < 2^{k_{1}+1} \vert z-z_{0}\vert } \bigl\vert K(z,y_{1},y_{2})-K(z_{0},y_{1},y_{2}) \bigr\vert \bigl\vert b_{1}(y_{1})-\lambda_{1} \bigr\vert \\ &{}\times \bigl\vert f_{1}(y_{1})\bigr\vert \bigl\vert b_{2}(y_{2})-\lambda_{2}\bigr\vert \bigl\vert f_{2}(y_{2})\bigr\vert \,dy _{1}\,dy_{2} \Biggr)^{\delta }\,dz \Biggr)^{\frac{1}{\delta }} \\ \leq & C \Biggl(\frac{1}{\vert B\vert } \int_{B} \Biggl( \sum_{k_{1}=1}^{\infty } \sum_{k_{2}=1}^{\infty } \int_{2^{k_{2}}\vert z-z_{0}\vert \leq \vert y_{2}-z_{0}\vert < 2^{k_{2}+1}\vert z-z_{0}\vert } \bigl\vert b_{2}(y_{2})- \lambda_{2}\bigr\vert \bigl\vert f_{2}(y_{2}) \bigr\vert \\ &{}\times \biggl( \int_{2^{k_{1}}\vert z-z_{0}\vert \leq \vert y_{1}-z_{0}\vert < 2^{k_{1}+1}\vert z-z_{0}\vert } \bigl\vert K(z,y_{1},y_{2})-K(z_{0},y_{1},y_{2}) \bigr\vert ^{q}\,dy_{1} \biggr)^{\frac{1}{q}} \\ &{}\times \biggl( \int_{2^{k_{1}+4}B} \bigl\vert b_{1}(y_{1})- \lambda_{1}\bigr\vert ^{q'} \bigl\vert f_{1}(y_{1})\bigr\vert ^{q'}\,dy_{1} \biggr)^{\frac{1}{q'}}\,dy_{2} \Biggr)^{\delta }\,dz \Biggr)^{\frac{1}{\delta }} \\ \leq & C \Biggl(\frac{1}{\vert B\vert } \int_{B} \Biggl( \sum_{k_{1}=1}^{\infty } \sum_{k_{2}=1}^{\infty } \biggl( \int_{2^{k_{1}+4}B} \bigl\vert b_{1}(y_{1})- \lambda_{1}\bigr\vert ^{q'} \bigl\vert f_{1}(y_{1})\bigr\vert ^{q'}\,dy_{1} \biggr)^{\frac{1}{q'}} \\ &{}\times \biggl( \int_{2^{k_{2}}\vert z-z_{0}\vert \leq \vert y_{2}-z_{0}\vert < 2^{k_{2}+1}\vert z-z_{0}\vert } \bigl\vert b_{2}(y_{2})- \lambda_{2}\bigr\vert ^{q'}\bigl\vert f_{2}(y_{2})\bigr\vert ^{q'}\,dy_{2} \biggr)^{ \frac{1}{q'}} \\ &{}\times \biggl( \int_{2^{k_{2}}\vert z-z_{0}\vert \leq \vert y_{2}-z_{0}\vert < 2^{k_{2}+1}\vert z-z_{0}\vert } \int_{2^{k_{1}}\vert z-z_{0}\vert \leq \vert y_{1}-z_{0}\vert < 2^{k_{1}+1}\vert z-z_{0}\vert } \bigl\vert K(z,y_{1},y_{2})\\ &{}-K(z_{0},y_{1},y_{2}) \bigr\vert ^{q}\,dy_{1}\,dy_{2} \biggr)^{\frac{1}{q}} \Biggr)^{\delta }\,dz \Biggr)^{\frac{1}{\delta }} \\ \leq & C \Biggl( \frac{1}{\vert B\vert } \int_{B} \Biggl( \sum_{k_{1}=1}^{\infty } \sum_{k_{2}=1}^{\infty } \biggl(\frac{1}{\vert 2^{k_{1}+4}B\vert } \int_{2^{k_{1}+4}B} \bigl\vert b_{1}(y_{1})- \lambda_{1}\bigr\vert ^{q'} \bigl\vert f_{1}(y_{1})\bigr\vert ^{q'}\,dy_{1} \biggr)^{\frac{1}{q'}} \\ &{}\times \biggl(\frac{1}{\vert 2^{k_{2}+4}B\vert } \int_{2^{k_{2}+4}B} \bigl\vert b_{2}(y_{2})- \lambda_{2}\bigr\vert ^{q'}\bigl\vert f_{2}(y_{2})\bigr\vert ^{q'}\,dy_{2} \biggr)^{\frac{1}{q'}} \bigl\vert 2^{k_{1}+4}B\bigr\vert ^{\frac{1}{q'}} \\ &{}\times \bigl\vert 2^{k_{2}+4}B\bigr\vert ^{\frac{1}{q'}} \vert z-z_{0}\vert ^{-\frac{2n}{q'}} C _{k_{1}}2^{-\frac{n}{q'}k_{1}} C_{k_{2}}2^{-\frac{n}{q'}k_{2}} \Biggr)^{ \delta }\,dz \Biggr)^{\frac{1}{\delta }} \\ \leq & C \Biggl( \frac{1}{\vert B\vert } \int_{B} \Biggl( \sum_{k_{1}=1}^{\infty } \sum_{k_{2}=1}^{\infty } \biggl(\frac{1}{\vert 2^{k_{1}+4}B\vert } \int_{2^{k_{1}+4}B} \bigl\vert b_{1}(y_{1})- \lambda_{1}\bigr\vert ^{q't'}\,dy_{1} \biggr)^{ \frac{1}{q't'}} \\ &{}\times \biggl(\frac{1}{\vert 2^{k_{1}+4}B\vert } \int_{2^{k_{1}+4}B} \bigl\vert f_{1}(y_{1})\bigr\vert ^{q't}\,dy_{1} \biggr)^{\frac{1}{q't}} \\ &{}\times \biggl(\frac{1}{\vert 2^{k_{2}+4}B\vert } \int_{2^{k_{2}+4}B} \bigl\vert b_{2}(y_{2})- \lambda_{2}\bigr\vert ^{q't'}\,dy_{2} \biggr)^{\frac{1}{q't'}} \\ &{}\times \biggl(\frac{1}{\vert 2^{k_{2}+4}B\vert } \int_{2^{k_{2}+4}B} \bigl\vert f_{2}(y_{2})\bigr\vert ^{q't}\,dy_{2} \biggr)^{\frac{1}{q't}} C_{k_{1}} C_{k_{2}} \Biggr)^{ \delta }\,dz \Biggr)^{\frac{1}{\delta }} \\ \leq & C \Vert b_{1}\Vert _{BMO} \Vert b_{2}\Vert _{BMO} M_{s}(f_{1}) (x) M_{s}(f_{2}) (x) \Biggl(\sum_{k_{1}=1}^{\infty }k_{1}C_{k_{1}} \Biggr) \Biggl(\sum_{k_{2}=1}^{\infty } k_{2}C_{k_{2}} \Biggr) \\ \leq & C \Vert b_{1}\Vert _{BMO} \Vert b_{2}\Vert _{BMO} M_{s}(f_{1}) (x) M_{s}(f_{2}) (x). \end{aligned}$$

Thus,

$$IV\leq C \Vert b_{1}\Vert _{BMO} \Vert b_{2}\Vert _{BMO} M_{s}(f_{1}) (x) M_{s}(f_{2}) (x). $$

Therefore,

$$\begin{aligned}& M^{\sharp }_{\delta }\bigl( T_{\Pi \vec{b}}(\vec{f})\bigr) (x) \\& \quad =M^{\sharp }\bigl( \bigl\vert T_{\Pi \vec{b}}(\vec{f})\bigr\vert ^{\delta }\bigr)^{\frac{1}{ \delta }}(x) \\& \quad \leq \sup_{B\ni x} \biggl( \frac{1}{\vert B\vert } \int_{B} \bigl\vert \bigl\vert T_{\Pi\vec{b}}(\vec{f}) (z)\bigr\vert ^{\delta }-\vert C_{0}\vert ^{\delta } \bigr\vert \,dz \biggr) ^{\frac{1}{ \delta }} \\& \quad \leq C \Vert b_{1}\Vert _{BMO} \Vert b_{2}\Vert _{BMO} \bigl( M_{s}(f_{1}) (x) M_{s}(f _{2}) (x)+M_{\varepsilon }\bigl(T(f_{1},f_{2}) \bigr) (x) \bigr) \\& \qquad {} + C \bigl(\Vert b_{1}\Vert _{BMO} M_{\varepsilon } \bigl(T_{b_{2}}^{2}(f_{1},f _{2})\bigr) (x)+\Vert b_{2}\Vert _{BMO} M_{\varepsilon } \bigl(T_{b_{1}}^{1}(f_{1},f_{2})\bigr) (x) \bigr), \end{aligned}$$

which completes the proof of Theorem 2.1. □

### Proof of Theorem 2.2

We have $w\in A_{\max \{\frac{p _{1}}{q_{1}},\ldots,\frac{p_{m}}{q_{m}}\}}\subset A_{\infty }$ from Lemma 3.4. By Lemma 3.6, for every $i=1,\ldots,m$, since $w_{i} \in A_{p_{i}/q'}$, there exists $l_{i}$ satisfying $1< l_{i}< p_{i}/q'$ and $w_{i} \in A_{l_{i}}$. Since $q'< p_{i}/l_{i}$, there exists $s_{i}$ satisfying $q'< s_{i}< p_{i}/l_{i}< p_{i}$. Let $s= \min_{1\leq i\leq m}s_{i}$, then we have $q'< s< p_{i}$. Since $l_{i}< p_{i}/s_{i}\leq p_{i}/s $, then $w_{i} \in A_{l_{i}} \subset A _{p_{i}/s}$, $i=1,\ldots,m$.

Choose $\delta, \varepsilon_{1}, \varepsilon_{2}, \ldots, \varepsilon_{m}$ satisfying $0<\delta <\varepsilon_{1}<\varepsilon _{2}<\cdots <\varepsilon_{m}<\frac{1}{m}$. It follows from Lemma 3.3 and Lemma 3.5 that

$$\bigl\Vert M_{\varepsilon_{j}}\bigl(T(\vec{f})\bigr)\bigr\Vert _{L^{p}(w)} \leq C\bigl\Vert M^{\sharp }_{\varepsilon_{j}}\bigl(T( \vec{f})\bigr)\bigr\Vert _{L^{p}(w)} \leq C \Biggl\Vert \prod _{i=1}^{m} M_{q'}(f_{i}) \Biggr\Vert _{L^{p}(w)},\quad j=1,\ldots,m. $$

By Theorem 2.1, we have

$$\begin{aligned}& \bigl\Vert M^{\sharp }_{\delta }\bigl( T_{\Pi \vec{b}}(\vec{f}) \bigr)\bigr\Vert _{L^{p}(w)} \\& \quad \leq C \prod_{j=1}^{m} \Vert b_{j}\Vert _{BMO} \Biggl( \Biggl\Vert \prod _{k=1}^{m}M_{s}(f_{k}) \Biggr\Vert _{L^{p}(w)} +\bigl\Vert M_{\varepsilon_{1}} \bigl( T(\vec{f})\bigr) \bigr\Vert _{L^{p}(w)} \Biggr) \\& \qquad {} + C \sum_{j=1}^{m-1} \sum _{\sigma \in C^{m}_{j}} \prod_{i=1} ^{j} \Vert b_{\sigma (i)}\Vert _{BMO} \bigl\Vert M_{\varepsilon_{1}} \bigl( T_{\Pi \vec{b}_{\sigma '}}(\vec{f})\bigr)\bigr\Vert _{L^{p}(w)} \\& \quad \leq C \prod_{j=1}^{m} \Vert b_{j}\Vert _{BMO} \Biggl( \Biggl\Vert \prod _{k=1}^{m}M_{s}(f_{k}) \Biggr\Vert _{L^{p}(w)} +\bigl\Vert M_{\varepsilon_{1}} \bigl( T(\vec{f})\bigr) \bigr\Vert _{L^{p}(w)} \Biggr) \\& \qquad {} + C \sum_{j=1}^{m-1} \sum _{\sigma \in C^{m}_{j}} \prod_{i=1} ^{j} \Vert b_{\sigma (i)}\Vert _{BMO} \bigl\Vert M^{\sharp }_{\varepsilon_{1}} \bigl( T_{\Pi \vec{b}_{\sigma '}}(\vec{f})\bigr)\bigr\Vert _{L^{p}(w)}. \end{aligned}$$

In order to reduce the dimension of $BMO$ functions in the commutators, we apply Theorem 2.1 again to $\Vert M^{\sharp }_{\varepsilon_{1}}( T_{\Pi \vec{b}_{\sigma '}}(\vec{f}))\Vert _{L^{p}(w)}$.

Let $\sigma = \{\sigma (1),\ldots,\sigma (j)\}$ and $\sigma '= \{ \sigma (j+1),\ldots,\sigma (m)\}$, $A_{h}=\{\sigma_{1}: \mbox{any finite subset of } \sigma ' \mbox{with different elements}\}$ and $\sigma '_{1}=\sigma '-\sigma_{1}$.

It follows from Theorem 2.1 that

$$\begin{aligned}& \bigl\Vert M^{\sharp }_{\varepsilon_{1}}\bigl( T_{\Pi \vec{b}_{\sigma '}}(\vec{f}) \bigr)\bigr\Vert _{L^{p}(w)} \\& \quad \leq C \prod_{k=j+1}^{m} \Vert b_{\sigma (k)}\Vert _{BMO} \Biggl( \Biggl\Vert \prod _{l=1}^{m}M_{s}(f_{l}) \Biggr\Vert _{L^{p}(w)} +\bigl\Vert M_{\varepsilon_{2}}\bigl( T(\vec{f})\bigr) \bigr\Vert _{L^{p}(w)} \Biggr) \\& \qquad {}+ C \sum_{h=1}^{m-j-1} \sum _{\sigma_{1}\in A_{h}} \prod_{i=1} ^{h} \Vert b_{\sigma_{1}(i)}\Vert _{BMO} \bigl\Vert M_{\varepsilon_{2}} \bigl( T_{\Pi\vec{b}_{\sigma '_{1}}}(\vec{f})\bigr)\bigr\Vert _{L^{p}(w)}. \end{aligned}$$

By putting the formula above into $\Vert M^{\sharp }_{\delta } ( T_{\Pi \vec{b}}(\vec{f}))\Vert _{L^{p}(w)}$, we can reduce the dimension of $BMO$ functions.

Repeating the process above and using Lemma 3.7, we can get

$$\begin{aligned}& \bigl\Vert M^{\sharp }_{\delta }\bigl( T_{\Pi \vec{b}}(\vec{f}) \bigr)\bigr\Vert _{L^{p}(w)} \\& \quad \leq C \prod_{j=1}^{m} \Vert b_{j}\Vert _{BMO} \Biggl( A_{m+1}(m,n) \Biggl\Vert \prod_{k=1}^{m}M_{s}(f_{k}) \Biggr\Vert _{L^{p}(w)} \\& \qquad {} +A_{1}(m,n)\bigl\Vert M_{\varepsilon_{1}} \bigl( T(\vec{f})\bigr) \bigr\Vert _{L^{p}(w)} +A _{2}(m,n)\bigl\Vert M_{\varepsilon_{2}}\bigl( T(\vec{f})\bigr) \bigr\Vert _{L^{p}(w)} \\& \qquad {} +\cdots +A_{m}(m,n)\bigl\Vert M_{\varepsilon_{m}} \bigl( T(\vec{f}) \bigr) \bigr\Vert _{L ^{p}(w)} \Biggr), \end{aligned}$$

where $A_{1}(m,n),A_{2}(m,n),\ldots,A_{m+1}(m,n)$ are finite real numbers related to *m* and *n*.

Then, by Lemma 3.3 and Lemma 3.5,

$$\begin{aligned} \bigl\Vert T_{\Pi \vec{b}}(\vec{f})\bigr\Vert _{L^{p}(w)} \leq& \bigl\Vert M_{\delta }\bigl( T_{\Pi \vec{b}}(\vec{f})\bigr)\bigr\Vert _{L^{p}(w)} \\ \leq& C\bigl\Vert M^{\sharp }_{\delta }\bigl( T_{\Pi \vec{b}}( \vec{f})\bigr)\bigr\Vert _{L^{p}(w)} \\ \leq& C \prod_{j=1}^{m} \Vert b_{j}\Vert _{BMO} \Biggl( A_{m+1}(m,n) \Biggl\Vert \prod_{k=1}^{m}M_{s}(f_{k}) \Biggr\Vert _{L^{p}(w)} \\ & {} +A_{1}(m,n)\bigl\Vert M_{\varepsilon_{1}} \bigl( T(\vec{f})\bigr) \bigr\Vert _{L^{p}(w)} +A _{2}(m,n)\bigl\Vert M_{\varepsilon_{2}} \bigl( T(\vec{f})\bigr) \bigr\Vert _{L^{p}(w)} \\ & {} +\cdots +A_{m}(m,n)\bigl\Vert M_{\varepsilon_{m}} \bigl( T(\vec{f}) \bigr) \bigr\Vert _{L ^{p}(w)} \Biggr) \\ \leq& C \prod_{j=1}^{m} \Vert b_{j}\Vert _{BMO} \Biggl( A_{m+1}(m,n) \Biggl\Vert \prod_{k=1}^{m}M_{s}(f_{k}) \Biggr\Vert _{L^{p}(w)} \\ & {} +A_{m+2}(m,n) \Biggl\Vert \prod_{j=1}^{m}M_{q'}(f_{j}) \Biggr\Vert _{L ^{p}(w)} \Biggr) \\ \leq& C \prod_{j=1}^{m} \Vert b_{j}\Vert _{BMO} \Biggl\Vert \prod _{j=1}^{m}M_{s}(f_{j}) \Biggr\Vert _{L^{p}(w)} \\ \leq& C \prod_{j=1}^{m} \Vert b_{j}\Vert _{BMO} \prod_{j=1}^{m} \bigl\Vert M_{s}(f_{j})\bigr\Vert _{L^{p_{j}}(w_{j})} \\ =& C \prod_{j=1}^{m} \Vert b_{j}\Vert _{BMO} \prod_{j=1}^{m} \bigl\Vert M\bigl(\vert f_{j}\vert ^{s}\bigr)\bigr\Vert ^{\frac{1}{s}}_{L^{\frac{p_{j}}{s}}(w_{j})} \\ \leq& C \prod_{j=1}^{m} \Vert b_{j}\Vert _{BMO} \prod_{j=1}^{m} \bigl\Vert \vert f_{j}\vert ^{s}\bigr\Vert ^{\frac{1}{s}}_{L^{\frac{p_{j}}{s}}(w_{j})} \\ =& C \prod_{j=1}^{m} \Vert b_{j}\Vert _{BMO} \prod_{j=1}^{m} \Vert f_{j} \Vert _{L ^{p_{j}}(w_{j})}, \end{aligned}$$

which completes the proof of Theorem 2.2. □

### Proof of Theorem 2.3

Let $q_{0}=\min_{1\leq j\leq m}q _{0}^{j}$, then $q'< q_{0}<\infty $. It follows from $p(\cdot)\in \mathcal{B}(\mathbf{R}^{n})$ and Lemma 3.11 that there exists $p_{0}$ satisfying $1< p_{0}< p_{-}$ and $( \frac{p(\cdot)}{p_{0}} ) ' \in \mathcal{B}(\mathbf{R}^{n})$.

Choose $\delta,\varepsilon_{1},\varepsilon_{2},\ldots,\varepsilon _{m}$ satisfying $0<\delta <\varepsilon_{1}<\varepsilon_{2}<\cdots < \varepsilon_{m}<\frac{1}{m}$. For any $w\in A_{1}$, we have

$$\begin{aligned} \int_{\mathbf{R}^{n}} \bigl\vert T_{\Pi \vec{b}}(\vec{f}) (x)\bigr\vert ^{p_{0}}w(x)\,dx =& \bigl\Vert T_{\Pi \vec{b}}(\vec{f})\bigr\Vert _{L^{p_{0}}(w)} ^{p_{0}} \\ \leq& \bigl\Vert M_{\delta }\bigl( T_{\Pi \vec{b}}(\vec{f})\bigr)\bigr\Vert _{L^{p_{0}}(w)} ^{p _{0}} \\ \leq& C \bigl\Vert M^{\sharp }_{\delta }\bigl( T_{\Pi \vec{b}}( \vec{f})\bigr)\bigr\Vert _{L^{p _{0}}(w)} ^{p_{0}} \\ \leq& C \Biggl( \prod_{j=1}^{m} \Vert b_{j}\Vert _{BMO} \Biggl( A_{m+1}(m,n) \Biggl\Vert \prod_{j=1}^{m}M_{q_{0}}(f_{j}) \Biggr\Vert _{L^{p_{0}}(w)} \\ & {} + A_{1}(m,n)\bigl\Vert M_{\varepsilon_{1}} \bigl(T(\vec{f})\bigr) \bigr\Vert _{L^{p_{0}}(w)}+ A_{2}(m,n)\bigl\Vert M_{\varepsilon_{2}} \bigl(T(\vec{f})\bigr) \bigr\Vert _{L^{p_{0}}(w)} \\ & {} +\cdots +A_{m}(m,n)\bigl\Vert M_{\varepsilon_{m}} \bigl( T(\vec{f}) \bigr) \bigr\Vert _{L ^{p_{0}}(w)} \Biggr) \Biggr)^{p_{0}} \\ \leq& C \Biggl(\prod_{j=1}^{m} \Vert b_{j}\Vert _{BMO} \Biggl( A_{m+1}(m,n) \Biggl\Vert \prod_{j=1}^{m}M_{q_{0}}(f_{j}) \Biggr\Vert _{L^{p_{0}}(w)} \\ & {} +A_{m+2}(m,n) \Biggl\Vert \prod_{j=1}^{m}M_{q'}(f_{j}) \Biggr\Vert _{L ^{p_{0}}(w)} \Biggr) \Biggr)^{p_{0}} \\ \leq& C \Biggl(\prod_{j=1}^{m} \Vert b_{j}\Vert _{BMO} \Biggl\Vert \prod _{j=1}^{m}M_{q^{j}_{0}}(f_{j}) \Biggr\Vert _{L^{p_{0}}(w)} \Biggr)^{p_{0}} \\ =& C \Biggl(\prod_{j=1}^{m} \Vert b_{j}\Vert _{BMO} \Biggr)^{p_{0}} \int_{\mathbf{R}^{n}} \Biggl(\prod_{j=1}^{m}M_{q^{j}_{0}}(f_{j}) (x) \Biggr)^{p_{0}} w(x)\,dx \end{aligned}$$

for all $\vec{f}=(f_{1},\ldots,f_{m})$ which are bounded measurable functions with compact support, where $A_{1}(m,n),A_{2}(m,n),\ldots,A _{m+2}(m,n)$ are finite real numbers related to *m* and *n*.

Applying Lemma 3.10 to $( T_{\Pi \vec{b}}(\vec{f}), \prod_{j=1}^{m}M _{q_{0}^{j}}(f_{j}))$, we have

$$\bigl\Vert T_{\Pi \vec{b}}(\vec{f})\bigr\Vert _{L^{p(\cdot)}(\mathbf{R}^{n})}\leq C \Biggl\Vert \prod_{j=1}^{m}M_{q_{0}^{j}}(f_{j}) \Biggr\Vert _{L^{p(\cdot)}( \mathbf{R}^{n})}. $$

Then it follows from Lemma 3.9 and Lemma 3.8 that

$$\bigl\Vert T_{\Pi \vec{b}}(\vec{f})\bigr\Vert _{L^{p(\cdot)}(\mathbf{R}^{n})} \leq C \prod_{j=1}^{m}\bigl\Vert M_{q_{0}^{j}}(f_{j})\bigr\Vert _{L^{p_{j}(\cdot)}(\mathbf{R} ^{n})} \leq C\prod _{j=1}^{m}\Vert f_{j}\Vert _{L^{p_{j}(\cdot)}(\mathbf{R} ^{n})}, $$

which completes the proof of Theorem 2.3. □
